# A Study of the Mechanism of the Congruence of Leader–Follower Power Distance Orientation on Employees’ Task Performance

**DOI:** 10.3389/fpsyg.2019.00615

**Published:** 2019-03-26

**Authors:** Yan Bao, Shudi Liao, Jianqiao Liao, Yucheng Zhang, Chuanjun Deng, Zhiwen Guo

**Affiliations:** ^1^School of Management, Huazhong University of Science and Technology, Wuhan, China; ^2^Business School, Hubei University, Wuhan, China; ^3^School of Economics and Management, Hebei University of Technology, Tianjin, China; ^4^Business School, Henan University, Kaifeng, China

**Keywords:** congruence of leader–follower power distance orientation, trust in supervisor, work engagement, task performance, power distance orientation

## Abstract

Based on implicit leadership theory, we examine the congruence effect of leader–follower power distance orientation (PDO) on follower trust in supervisor and work engagement, which in turn influences employees’ task performance. Results of polynomial regressions on 526 dyads supported the congruence effect hypothesis. The results show that (1) the congruence of leader–follower PDO leads to better performance; (2) under the condition of congruence, subordinate task performance is higher when leader–follower PDO matching in low–low ratings congruence than it is in high–high ratings congruence; (3) under the condition of asymmetrical incongruence, the follower had higher task performance when a leader’s PDO is lower than a follower’s PDO; (4) trust in supervisor and the work engagement mediate the effect of congruence of leader–follower PDO on employees’ task performance; (5) trust in supervisor also mediates the effect of congruence of leader–follower PDO on employees’ work engagement.

## Introduction

Power distance reflects the acceptance of the unequal distribution of power in a society, and individuals often show different power distance orientation (PDO). Since Hofstede (2001) discussed the concept of power distance proposed by Mulder (1976) at the national level in the 1980s, the academic circle has never stopped discussing this concept. Nowadays, on the one hand, the development of the internet has brought the change of social power structure. On the other hand, millennials with a high sense of individual power enter the workplace. All these make the difference between the leader and the subordinate in the power distribution and the status cognition of the Leader and the subordinate in organization show the characteristics of different times. However, from the existing studies, although previous studies have made many breakthroughs and laid a good foundation for the study of power distance in the organizational context, there are still the following limitations:

First, although the effect of power distance has covered the country, the organization, team and individual four aspects, only at the national level research thorough. The influence of the organization, team and individual level is limited, especially the relationship between the individual PDO and individual output remains to be further explored. Secondly, at present, the discussion on the mediating mechanism of power distance and its influence effect at all levels are extremely limited. The research at the organizational level mainly focuses on the relevant work variables, while the discussion at the individual level mostly focuses on the psychological level. Insufficient attention has been paid to other variables, especially the lack of relevant research on the mechanism of power distance on the individual behavior of employees. Finally, previous researches on PDO at the individual level mostly treated leaders and subordinates as independent individuals, but the discussion on the congruence of PDO on the leader and follower and the corresponding influence mechanism are still not systematic explored enough (Cole et al., 2013; Graham et al., 2018).

Based on implicit leadership theory, this study attempts to addresses this issue by using polynomial regression and surface response analysis to analyze the congruence/incongruence effect and mediation effect of leadership-subordinate PDO on employees’ task performance. The study aims to extend the current research on leader–follower relation, and benefits to organizations for improving the internal management effectiveness of enterprises.

## Theory and Hypothesis

### Matching Types of Leader-Subordinate Power Distance Orientation

By dividing the individual PDO of leaders and subordinates into high and low category, four types of leader–follower PDO can be obtained as shown in Table 1. The matching types include low–low ratings congruence and high–high ratings congruence, while mismatching types include high–low rating incongruence and low–high rating incongruence. In this study, we explore whether the congruence effect of leadership-follower PDO promote employees’ task performance, and the different effect between the high rating matching and low rating matching. In addition we also test the difference between “leader-high rating, subordinate-low rating,” and “leader-low rating, subordinate-high rating” in PDO under the mismatched situation. Table 1 shows the comparison of leader PDO and follower PDO in four categories.

**Table 1:** Comparison of leader power distance orientation (PDO) and follower PDO in four categories.

	Leader power distance orientation	
	Low power distance orientation	High power distance orientation
	Emphasizing equality, tend to think of the relationship between superior and subordinate as an equal mode of cooperation.	Emphasizing hierarchical, tend to think of the relationship between superiors and subordinates as obedience and being obeyed.

**Follower power distance orientation**		

**High power distance orientation.**	**Low–high rating incongruence.**	**High–high ratings congruence**

Accept hierarchical and power difference between leaders and subordinates.	While the leader recognizes the independent status of the employees, the employees also respect the authority of the leaders, and recognize the differential distribution of power among the subordinates in the organization.	The leader does not recognize the independent status of the employees, but the employees respect the authority of the leaders and recognize the difference in the distribution of power among the subordinates in the organization.
**Low power distance orientation**	**Low–low ratings congruence**	**High–low rating incongruence**
Emphasizing equality, ignoring power differences between leaders and subordinates.	While leaders recognize the independent status of employees, employees do not recognize the differential distribution of power within organization.	While leaders do not recognize the independent status of employees, employees also do not recognize the differential distribution of power within organization.

### Research Basis and Theoretical Hypothesis

Power distance orientation refers to the degree to which individuals accept the unequal distribution of power in society and organizations (Clugston et al., 2000). This reflects individuals’ perceptions of their position, authority, and power in an organization, and has an impact on leadership style and employees’ cognition and behavior (Kirkman et al., 2009). Trust in supervisor indicates that employees have positive expectations of their immediate supervisors and are willing to bear losses and to identify with the supervisors’ behavior under uncertain circumstances (Dirks, 2000). Work engagement is the continuous and positive emotional state that employees may show in terms of their work, and has the three characteristics of vitality, dedication, and focus. Vitality expresses the abundant energy and good psychological resilience of staff in their work, dedication is reflect the staff’s high level of engagement in their own work and full enthusiasm for it, and focus refers to their positive cognitive experience of immersion in work (Schaufeli et al., 2006).

According to implicit leadership theory, employees in an organization make effective or ineffective judgments of their leader’s behavior based on the particular preconceptions of leadership they hold (Eden and Leviatan, 1975). When the perceived specific leadership traits and behaviors are consistent with their implicit leadership expectations, their cognitive schemata will be activated (Schyns and Schilling, 2011) and they will respond positively to leaders’ behavior, which in turn leads to positive emotions and attitudes at work. such as trust in supervisor and work engagement.

Specifically, when the leader–follower PDO is congruent, both leader and follower have agreement on the distribution of organizational power and the different status of supervisor and subordinate. Under this circumstance, the leadership style is consistent with the subordinate’s implicit expectations. In addition, when the PDO of leader and follower both high rating, even if the high power distance-oriented leadership prefers centralized management (Low et al., 2011), high power distance-oriented employees will obey the instructions of the leaders without any objection (Cole et al., 2013). leadership style with a high power distance can be regarded as “wise” and “undeniable” in the eyes of leaders themselves and subordinates, and employees may have high levels of trust in supervisors due to their high levels of respect and more likely to be engaged in real work because they recognize the inspiring personal charm of their leaders (Avolio et al., 1999). With the corresponding, when the PDO of both leader and follower are low, leaders with low levels of PDO recognize the independent status of employees and pay attention to communicating with subordinate employees (Kirkman and Shapiro, 2001). This leadership style is considered as “amiable” by both leaders and subordinates. Employees may have high levels of trust in supervisors due to their feel approachable of their leader. We thus predict higher levels of trust in supervisors for employee when leader and follower have matched, as opposed to mismatched in PDO. At the same time, high-quality communication and interaction between superiors and subordinates will also leads to positive emotional experience for employees and put them into the work wholeheartedly (Strom et al., 2014).

H1a Congruence between leader and follower PDO will be positively related to employee trust in supervisor.
H1b Congruence between leader and follower PDO will be positively related to employee work engagement.

Brown et al. (2005) show that leaders with low PDO consider subordinates’ opinions more in decision-making, so the employees expect to participate in decision-making under the matching model of leader–follower low PDO. Their enthusiasm to take the initiative is then recognized by leaders, thus generating strong work motivation. For example, Cordery et al. (2010) find that employees who participate in decision-making have stronger internal motivation. In addition, the frequent communication between superiors and subordinates makes low power distance-oriented employees feel closer. The research of Gulati and Sytch (2008) also shows that frequent communication between leaders and followers is positively related to mutual trust. It can be seen that under the condition of low–low ratings congruence, the trust in supervisor and work engagement of employees’ are generated with a lot of initiative motivations. However, under the leader–follower high power distance-oriented matching model, employees are found to be motivated by reverence, and the “wisdom” of leaders is respected by employees, while their “unquestionable” status makes employees more afraid of them out of respect, and they fear that they will be punished by leaders for not working hard. The generation of power is therefore passive. Accordingly, we hypothesize that:

H2a Subordinate trust in supervisor is higher when leader–follower PDO matching in low–low ratings congruence than it is in high–high ratings congruence.
H2b Subordinate work engagement is higher when leader–follower PDO matching in low–low ratings congruence than it is in high–high ratings congruence.

The implicit image of leaders in the minds of employees is found to be significantly influenced by cultural factors (Den Hartog et al., 1999). Unlike the values of individualism, freedom, and equality advocated in Western societies, the hierarchical tradition in Eastern societies have led to social and cultural characteristics such as high power distance, patterns of difference, and respect for authority. Leaders are often regarded as having higher status, and gaining the recognition and respect of leaders will thus stimulate more internal motivation in employees. Approachable leaders who exhibit low power distance-oriented leadership make employees with high PDO feel more comfortable, rather than in awe. Accordingly, the following hypotheses are put forward.

H3a Trust in supervisor is higher when a leader’s PDO is lower than a follower’s PDO.
H3a Work engagement is higher when a Leader’s PDO is lower than a follower’s PDO.

### The Mediating Effect of Trust in Supervisor and Work Engagement on Power Distance Orientation Congruence and Employee Task Performance

Studies have shown that trust in supervisors has a positive effect on employees’ task performance (Dirks and Ferrin, 2002; Mayer and Gavin, 2005). According to the trust model proposed by Mayer et al. (1995), supervisor trust reflects subordinates’ evaluations of leaders’ reliability through daily communications. For example, Mach and Lvina (2016) showed that trust in leaders can be converted into trust in the team, which has a positive impact on team performance. The reason is that trust can enhance employees’ psychological security and focus their attention. Therefore, based on the combination of trust theory and implicit theory, this study attempts to open the “black box” between the leader-subordinate PDO congruence and work performance, namely, to explore the mediating mechanisms of trust in supervisor. Specifically, when employees have a high level of trust in supervisors, it means that employees have a strong sense of psychological security (Huang et al., 2010). They believe that supervisors will not abuse their power and harm the interests of the employees, and the employees will then be more willing to obey the supervisors and support their decision-making (Sanchez, 2002). When employees feel that a supervisor is trustworthy, they will also emotionally identify with the supervisor, and be willing to spend more energy to complete the tasks assigned by the supervisor (Ellis, 2015). Our first three hypotheses address the influence of leader-subordinate power distance-oriented congruence on employee trust in supervisor. Considering the positive correlation between trust in supervisor and task performance, we argue that trust in supervisor plays a mediating role between leader-subordinate power distance-oriented congruence and employee task performance. Accordingly, we present the following hypothesis:

H4 Trust in supervisor mediates the relationship between the leader–follower PDO congruence and employee task performance.

Work engagement is the basis of task performance. According to Kahn (1990), highly engaged employees devote themselves to their work with a high degree of vitality and dedication, and thus achieve higher task performance. Parker et al. (2010) demonstrated the relationship between work engagement and performance, and argued that employees with high levels of work engagement are more likely to get recognition and support from leaders and organizations. Consequently, they have more social resources to achieve performance goals which expected in employees’ work than employees with low levels of work engagement. The first three hypotheses consider the impact of congruence in leader–follower PDO on work engagement. For example, when leader–follower PDO are congruent, leadership behavior will meet employees’ implicit leadership cognition. No matter in the eyes of leadership itself or subordinates, leaders demonstrated leadership style is considered right and encouraging, the positive emotional experience could prompt employees to more actively into work (Strom et al., 2014). When leader–follower PDO is incongruent, there is a difference between the leadership behavior and employees’ implicit cognition, which makes the employees often need to spend extra effort to adapt and understand the behavior intention of leadership, thus reducing their work engagement. Combined with the positive correlation between work engagement and task performance, we argue that work engagement plays a mediating role between leader–follower PDO congruence and employee task performance. Accordingly, we present the following hypothesis:

H5 Work engagement mediates the relationship between the leader–follower PDO congruence and employee task performance.

### The Mediating Role of Trust in Supervisor Between Leader–Follower Power Distance Orientation Congruence and Employee Work Engagement

**FIGURE 1:**
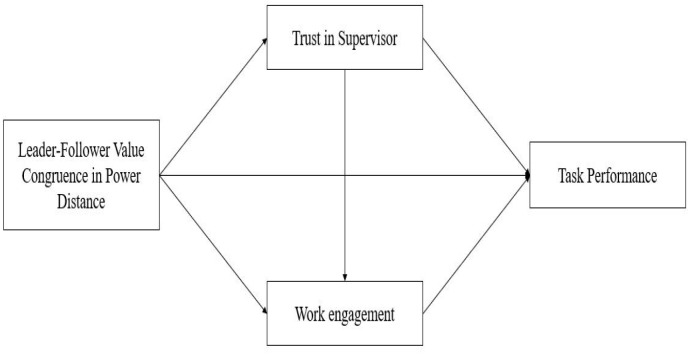
Conceptual model.

Most studies show that trust has a positive impact on work engagement. Chughtai and Buckley (2008) suggest that when employees trust direct leadership, they are more confident that they can get effective help from leaders when they encounter work-related obstacles, while Schaufeli and Bakker (2004) show that employees’ trust in direct leadership makes them aware of the leader. They have the resources to achieve the goals required, resulting in greater input to their work. Trust in leadership also means that employees think they will be treated fairly in the organization. Albrecht (2010) indicates that there is a positive relationship between perceived organizational fairness and employee work engagement. Combining the first three hypotheses, we argue that trust in supervisor plays a mediating role between leader–follower PDO congruence and employee work engagement. Accordingly, we present the following hypothesis:

H6 Trust in a supervisor mediates the relationship between leader–follower PDO congruence and employee work engagement. The conceptual model of this study is as follows (see Figure 1):

## Materials and Methods

### Participants and Procedures

We collected data from 12 enterprises in the People’s Republic of China. These enterprises are from various industries, including machinery manufacturing, IT, education, and retail. We collected data at two-phase survey in order to avoid the contamination of Common Source Bias. Firstly, we assigned every participant a unique identification code with the cooperation of the human resources department. There are corresponding codes on the questionnaires received by employees, while leaders need to fill in the names of subordinates when they receive the questionnaires to evaluate subordinates. Leaders do not need to fill in their names during the entire filling process to ensure their anonymity. At time 1, we sent questionnaires to 600 employees and 130 leaders. In this stage, we measured the leader and follower PDO, respectively. A month later, we carried out the second phase of the survey, at time 2, we measured the trust in supervisor and work engagement which are reported by subordinates. In addition, leaders rated employees’ task performance. After the questionnaires were collected, the missing and unmatched questionnaires were excluded. In total, 526 employee questionnaires and 121 leadership questionnaires were finally obtained, with the response rate of 87.7 and 93.1%, respectively. On average, one leader has 4.3 subordinates.

In the paired samples, the demographic characteristics of direct leadership were as follows: male managers accounted for 66.9%, female managers accounted for 33.1%, the average age was 35.26 (standard deviation 6.07), and the average education years was 16.27 (standard deviation 2.36). The population characteristics of subordinates are as follows: 37.8% male, 62.2% female, average age is 30.87 years old (standard deviation 5.70), average working years in this enterprise are 4.51 years, average tenure with the supervisor are 2.83 years (standard deviation 2.63), average years of education is 15.27 years (standard deviation 2.37).

#### Ethics Statement

We have consulted Huazhong University of Science and Technology’s ethics committee approval to conduct this survey before starting to collect data. The ethics committee of the university has reviewed and approved our study. This study does not violate any legal regulations or common ethical guidelines based on our research design. We introduced our research purpose, goals and plans to each participants and asked their permission to participate in this academic research, which further ensured ethical innocuousness during our data collection in 12 companies. All written informed consent from the supervisors and employees were implied through survey completion.

### Measures

Likert 7-point scale was adopted in all scales in this study, and the scale ranging from 1 (totally disagree) to 7 (totally agree) unless otherwise specified. The method of back-translation (Brislin, 1980) was adopted to translate the scale into Chinese (e.g., Zhang et al., 2016, 2017). The scales used in this study has been tested in Chinese organizational contexts and have shown good reliability and validity (Schaubroeck et al., 2011). We detail the variables in the following sections.

#### Power Distance Orientation

We used a six-item measure of power distance developed by Dorfman and Howell (1988). Sample items are “Managers should make most decisions without consulting subordinates” and “It is frequently necessary for a manager to use authority and power when dealing with subordinates.” In this study, coefficient alpha was 0.779 for leaders and 0.795 for followers.

#### Trust in Supervisor

We used a five-item measure of trust in supervisor developed by Podsakoff et al. (1990). Sample items are “I feel quite confident that my leader will always try to treat me fairly.” and “I have complete faith in the integrity of manager/supervisor.” In this study, coefficient alpha was 0.863.

#### Work Engagement

Followers’ work engagement was measured using the nine-item adapted from Schaufeli et al. (2006). Sample items are “At my work, I feel bursting with energy” and “I am enthusiastic about my job.” In this study, coefficient alpha was 0.955.

#### Task Performance

We measured task performance by the five-item scale developed by Bachrach et al. (2007). Sample items are “Adequately complete assigned duties” and “Fulfill responsibilities specified in the job description.” In this study, coefficient alpha was 0.885.

#### Control Variables

According to the Cole et al. (2013) and Matta et al. (2015), we controlled for demographic similarity (that is, gender similarity, age similarity, and education similarity), because similarity would influence the development of individual PDO. Gender similarity is operated with dummy variables, with similar coded as “1” and dissimilar coded as “0.” Age similarity and education similarity was expressed using absolute differences in the age and the years of education of supervisors and subordinates. According to the advice of Cole et al. (2013), we also controlled for tenure (i.e., length of individual members’ exposure to a leader), because of the possibility that the tenure with the supervisor might affect leader–follower relationships and employee performance levels.

### Analysis

#### Polynomial Regressions

This study uses polynomial regression and response surface methodology to examine the congruence effect of leader-subordinate PDO (Edwards, 2002). According to the research of Edwards and Cable (2009), the following formula is constructed (to simplify, we omitted all control variables):

$$Z=\text{b}0+\text{b}1\;(L)+\text{b}2\;(F)+\text{b}3\;(L^{2})+\text{b}4\;(F\times L)+\text{b}5\;(F^{2})+e$$

In this equation, *Z* represents the dependent variable (viz., Trust in supervisor/Work engagement), and *L* and *F* are leader and follower PDO, respectively. Then we use the regression coefficients to draw the three-dimensional response surfaces, where *L* and *F* are drawn on the perpendicular horizontal axes, and *Z* is drawn on the vertical axis (Edwards and Parry, 1993). According to the research of Edwards and Cable (2009), we will test hypotheses 1, 2, and 3, respectively, by using three key characteristics of response surface that provide evidence for a congruence effect.

The first feature involves the curvature of the incongruence line, and this feature is necessary to test the congruence effect (i.e., Hypothesis 1a and 1b). Secondly, the slope (calculated as b1 + b2) and curvature (calculated as b3 + b4 + b5) along the congruence line is used to test H2a and H2b. Finally, the surface slope along the incongruence line (calculated as b1 − b2) and the lateral shift (calculated as [b2 − b1]/[2^∗^(b3 − b4 + b5)]) will be used to examine H3a and H3b (Edwards and Parry, 1993; Atwater et al., 2010).

#### Block Variable

This study uses polynomial regression coefficient to establish block variables (Edwards and Cable, 2009) to test the mediation hypotheses (H4, H5, and H6). Specifically, block variable is the weighted linear combination of the five polynomial terms: *L* (leader PDO), *F* (subordinate PDO), *L*^2^ (leading PDO square), *L* × *F* (the product of the leader PDO and subordinates PDO), *F*^2^ (subordinate PDO squared). This study used block variables as show congruence/incongruence effects of independent variables to test the mediation effect exists.

## Results

### Confirmatory Factors Analyses

This study uses confirmatory factors analyses to confirm each variable has good measurement validity, including the PDO of subordinate, trust in supervisor, work engagement and employee task performance, respectively. The results show that the five-factor model has fitted well (χ^2^ = 1165.96, df = 424, GFI = 0.87, CFI = 0.92, TLI = 0.91, RMSEA = 0.06). Moreover, the goodness of fit of this model is significantly higher than that of the other four alternative models. Table 2 shows the results of model fit comparisons.

**Table 2:** Model fit results for confirmatory factor analyses^a^.

MODEL	χ^2^	df	χ^2^/df	RMSEA	GFI	CFI	TLI
Five-factor model	1165.96	424	2.75	0.06	0.87	0.92	0.91
Four-factor model	1908.66	428	4.46	0.08	0.77	0.84	0.82
Three-factor model	2618.96	431	6.08	0.10	0.70	0.76	0.74
Two-factor model	3555.69	433	8.21	0.12	0.63	0.66	0.63
Single-factor model	4701.52	434	10.83	0.14	0.54	0.53	0.50

*^a^N = 526; four-factor model, power distance orientation of leader and power distance orientation of subordinate are combined; three-factor model, power distance orientation of leader, power distance orientation of subordinate and trust in supervisor are combined; two-factor model, power distance orientation of leader, power distance orientation of subordinate, trust in supervisor and work engagement are combined; single-factor model combines all items.*

In addition, since the mediating variables of this study (trust in supervisor and work engagement) were reported by employees themselves in the second stage of the survey, the common method variance might be caused. Thus, we used Harman’s single-factor test, which was regarded by Podsakoff and Organ (2016) as an *post hoc*-remedy method to avoid the homologous error problem. The results showed that the six factors extracted from the factor analysis explained 66.18% of the total variance, and the first factor explained 27.14% of the total variance, accounting for less than 50% of the total variance.

### Descriptive Statistical Analysis

Table 3 shows the means, standard deviations, inter-correlations, and reliability coefficients of the variables. It can be seen that trust in supervisor is significantly positively correlated with task performance (*R* = 0.30, *P* < 0.001); work engagement is significantly positively correlated with task performance (*R* = 0.32, *P* < 0.001); trust in supervisor and work engagement are significantly positive correlation (*R* = 0.41, *P* < 0.01).

**Table 3:** Means, standard deviations, and intercorrelations among study variables.

		*M*	*SD*	1	2	3	4	5	6	7	8
(1) Gender similarity		0.47	0.50	1							
(2) Age similarity		7.00	5.52	0.00	1						
(3) Education similarity		1.64	1.64	−0.01	−0.01	1					
(4) Tenure with the supervisor		2.83	2.63	0.10^∗^	0.02	−0.02	1				
(5) Power distance orientation of leader		3.68	0.90	−0.08	0.10^∗^	0.18^∗∗∗^	0.03	1			
(6) Power distance orientation of follower		3.60	1.05	0.05	0.06	0.19^∗∗∗^	0.01	0.12^∗∗^	1		
(7) Trust in supervisor		5.29	0.99	−0.01	0.03	−0.03	0.01	−0.18^∗∗∗^	0.04	1	
(8) Work engagement		4.95	1.08	−0.04	−0.03	−0.04	−0.03	−0.14^∗∗^	−0.02	0.41^∗∗^	1
(9) Task performance		4.79	1.13	0.05	0.04	−0.04	0.11^∗^	−0.29^∗∗∗^	−0.01	0.30^∗∗∗^	0.32^∗∗∗^

*^∗^p < 0.05, ^∗∗^p < 0.01, ^∗∗∗^p < 0.001.*

### The Congruence Effect of Leader–Follower Power Distance Orientation on Trust in Supervisor and Work Engagement

In order to test this effect, this study used the trust in supervisor and work engagement as the dependent variables to carry out the multiple regression separately: *L* (the first term of the leadership power distance) and *F* (the first term of the subordinate power distance), *L*^2^ (the square of the leadership power distance), *F* × *L* (the product of the leadership power distance and the subordinate power distance), and *F*^2^ (the square of the subordinate power distance).

According to the regression results in Table 4, after adding the square-item and interaction term of the leader-subordinate PDO, the explained variance of trust in supervisor and task performance in model 3 was significantly increased (Δ*R*^2^ = 0.06, *P* < 0.01; Δ*R*^2^ = 0.04, *P* < 0.05); Model 6 also appeared the same significantly increased (Δ*R*^2^ = 0.02, *P* < 0.05). This significant change in *R*^2^ indicates that there is a non-linear relationship between leader–follower PDO and trust in supervisor and work engagement. Therefore, it is more significant to analyze the outcome variables through a congruence method (Edwards, 2002; Cole et al., 2013; Carter and Mossholder, 2015).

**Table 4:** Polynomial regressions of trust in supervisor, work engagement and task performance on leader–follower power distance orientation congruence.

Variables	Trust in supervisor			Work engagement				Task performance				
	Model 1	Model 2	Model 3	Model 4	Model 5	Model 6	Model 7	Model 8	Model 9	Model 10	Model 11	Model 12
Constant	5.28^∗∗∗^	5.25^∗∗∗^	5.32^∗∗∗^	5.10^∗∗∗^	5.06^∗∗∗^	5.11^∗∗∗^	2.84^∗∗∗^	4.62^∗∗∗^	4.53^∗∗∗^	4.60^∗∗∗^	3.31^∗∗∗^	3.18^∗∗∗^
Gender similarity	−0.02	−0.05	−0.03	−0.09	−0.12	−0.11	−0.09	0.08	0.02	0.04	0.05	0.07
Age similarity	0.01	0.01	0.01	−0.01	0.00	0.00	−0.01	0.01	0.01	0.01	0.01	0.02
Education similarity	−0.02	0.00	−0.01	−0.03	−0.01	−0.01	−0.01	−0.03	0.01	0.00	0.00	0.00
Tenure with the supervisor	0.00	0.01	0.01	−0.01	−0.01	0.00	−0.01	0.04^∗^	0.05^∗∗^	0.05^∗^	0.05^∗^	0.05
Power distance orientation of leader (L)		−0.21^∗∗∗^	−0.19^∗∗∗^		−0.17^∗^	−0.16	−0.07		−0.38^∗∗∗^	−0.36^∗∗∗^	−0.32^∗^	−0.32^∗∗∗^
Power distance orientation of follower (F)		0.05	0.05		0.01	0.00	−0.02		0.02	0.02	0.01	0.02
*L*^2^			−0.12^∗∗^			−0.08	−0.03			−0.11	−0.08	−0.08^∗^
*F* ∗*L*			0.23^∗∗∗^			0.13^∗^	0.03			0.21^∗∗∗^	0.16^∗^	0.18^∗∗∗^
*F*^2^			−0.01			0.00	0.00			−0.01	−0.01	−0.01
ST							0.43^∗∗∗^				0.24^∗∗∗^	
WE												0.28^∗∗∗^
*R*^2^	0.00	0.04	0.10	0.01	0.02	0.04		0.02	0.10	0.14		
Adjust *R*^2^	−0.01	0.03	0.09	0.00	0.01	0.03		0.01	0.09	0.13		
Δ*R*^2^			0.06^∗∗^			0.02^∗^				0.04^∗^		
*F*	0.28	3.44^∗^	5.81^∗∗∗^	0.60	2.38	2.86^∗^		2.10	9.89^∗∗∗^	9.63^∗∗∗^		
Congruence (*L* = *F*)												
Slope (b1 + b2)			−0.15^∗^			−0.16^∗∗∗^				−0.35^∗∗∗^		
Curvature (b3 + b4 + b5)			0.10^∗^			0.05				0.10		
Incongruence (*L* = −*F*)												
Slope (b1 − b2)			−0.24^∗∗∗^			−0.15^∗^				−0.38^∗^		
Curvature (b3 − b4 + b5)			−0.36^∗∗∗^			−0.22^∗^				−0.33^∗^		
Lateral shift [b2 − b1]/[2 ∗ (b3 − b4 + b5)]			−0.33			−0.34				−0.58		

*^∗^p < 0.05, ^∗∗^p < 0.01, ^∗∗∗^p < 0.001; Unstandardized regression coefficients are reported. ST, trust in supervisor; WE, work engagement.*

According to the polynomial regression and response surface analysis results of Model 3 and Model 6 in Table 4.The surface along the incongruence line (*L* = −*F*) exhibits an inverted U-shape with a significant downward curvature (For trust in supervisor: curvature = −0.36, *P* < 0.001; For trust in supervisor: curvature = −0.22, *P* < 0.05), which indicates that trust in supervisor and work engagement is higher when leader and follower are aligned at a high level of PDO than that when leader and subordinate PDO are aligned at a low level. Thus supporting Hypothesis 1a and 1b. The slope of the congruence line (*L* = *F*) is significant and negative (For trust in supervisor: slope = −0.15, *P* < 0.05; For work engagement: slope = −0.16, *P* < 0.001), while the curvature along the congruence line is curved upward (For trust in supervisor: curvature = 0.10, *P* < 0.5; For work engagement: curvature = 0.05, *P* = 0.370). This indicating that the low–low congruence condition has higher trust in supervisor and work engagement than the high–high congruence condition. the hypothesis 2a and hypothesis 2b is supported. The slope along the incongruence line (*L* = −*F*) is significant negative (For trust in supervisor: slope = −0.24, *P* < 0.001; For work engagement: slope = −0.15, *P* < 0.05), and the lateral shift is negative (For trust in supervisor: lateral shift = −0.33; For work engagement: lateral shift = −0.34) indicating the asymmetrical incongruence effect of leader–follower PDO, Thus, when the leader’s PDO is lower than the follower’s, trust in supervisor and work engagement than it does when the leader’s PDO is higher than his/her follower’s supporting Hypothesis 3a and 3b.

**FIGURE 2:**
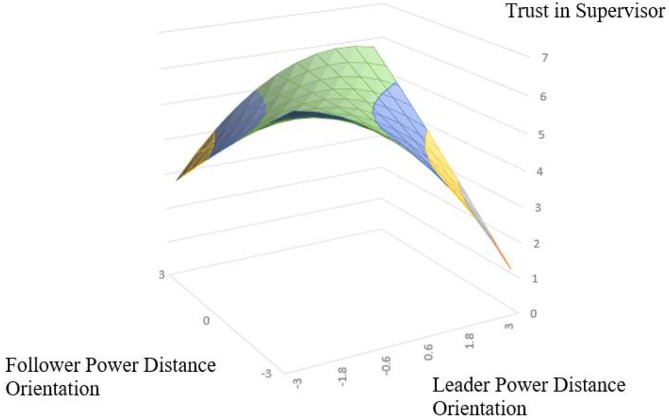
Congruence effect and asymmetrical incongruence effect of leader and follower power distance orientation on trust in supervisor.

**FIGURE 3:**
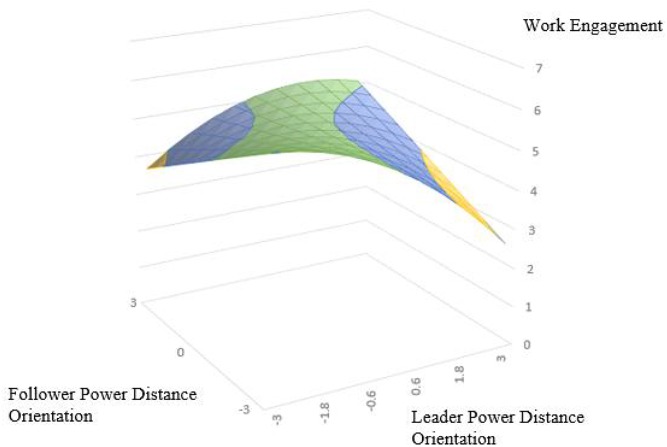
Congruence effect and asymmetrical incongruence effect of leader and follower power distance orientation on work engagement.

In order to interpretation of the results, we make the response surface based on these coefficients, as shown in Figure 2, 3. In Figure 2, 3, the congruence line is from the front corner (where *L* = *F* = −3.0) to the rear corner (where *L* = *F* = 3.0). whereas the incongruence line is from the left corner (where *L* = −3.0, *F* = 3.0) to the right corner (where *L* = 3.0, *F* = −3.0). As shown in Figure 2, the trust in supervisor is higher in the rear corner (higher in the green position, *R* = 6.62), and the trust in supervisor is higher in the left corner (leader-low, subordinate-high, *R* = 2.75) than the right corner (leader-high, subordinate-low, *R* = 1.34). As shown in Figure 3. The work engagement is higher in the rear corner (higher in the green position, *R* = 6.02) and the trust in supervisor is higher in the left corner (leader-low, subordinate-high, *R* = 3.64) than the right corner (leader-high, subordinate-low, *R* = 2.71). Response surface analysis and polynomial regression analysis showed consistent results.

### The Mediating Effect of Trust in Supervisor on the Relationship Between Leader and Follower Power Distance Orientation Congruence/Incongruence and the Task Performance

**FIGURE 4:**
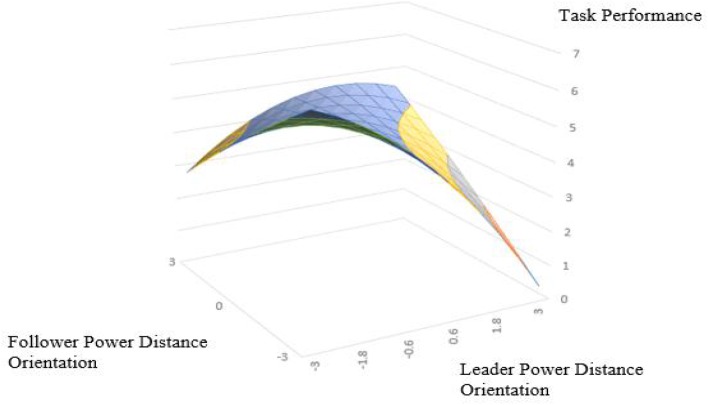
Congruence effect and asymmetrical incongruence effect of leader and follower power distance orientation on task performance.

Hypothesis 4 suggest the effect of leader and follower PDO congruence on task performance is transmitted through trust in supervisor. We performed polynomial regression with the task performance as the dependent variable again (the results are shown in model 9 in Table 3). As show in response surface analysis, the surface along the incongruence line (*L* = −F) in Figure 4 exhibits an inverted U-shape with a significant downward curvature (curvature = −0.33, *P* < 0.05). We found the slope of the congruence line (*L* = *F*) is negative (slope = −0.35, *P* < 0.001), and along the incongruence line (*L* = −*F*) is negative and significant (slope = −0.38, *P* < 0.05), which indicates that task performance increase as leader and follower PDO become more aligned, and the task performance is better when leader-subordinate PDO is congruence in low–low matching condition than when leader-subordinate PDO is congruence in high–high matching condition. In addition, when the leader’s PDO is lower than the follower’s, task performance is higher than it does when the leader’s PDO is higher than his/her follower’s. This is consistent with the regression results of trust in supervisor and work engagement.

In this study, block-variable approach is used to verify the mediation effect (H4 and H5) (Edwards and Cable, 2009; Zhang et al., 2012). According to the research of Lambert et al. (2006). We use polynomial coefficients to construct block variables representing the congruence effect. The results was shown in Tables 5, 6:

**Table 5:** Results for indirect effects of leader and follower power distance orientation congruence on task performance.

Variable	Mediator trust in supervisor	Task performance
Coefficient of the block variable	0.33 (a path)	0.28^∗∗∗^ (direct effect)
Trust in supervisor	0.24 (b path)	
Indirect effect (a∗b) of congruence via TS		0.08^∗∗^ (indirect effect)
99% bootstrapped CIs for indirect effect (ab)		(0.04, 0.13)

*Standardized coefficients are reported. Bias corrected confidence intervals (CIs) in 20,000 bootstrap samples are reported. Control variables include gender similarity, age similarity, education similarity, and tenure with the supervisor. TS, Trust in supervisor. ^∗^p < 0.05, ^∗∗^p < 0.01, ^∗∗∗^p < 0.001.*

**Table 6:** Results for indirect effects of leader and follower power distance orientation congruence on task performance.

Variable	Mediator work engagement	Task performance
Coefficient of the block variable (a path)	0.20 (a path)	0.32^∗∗∗^ (direct effect)
Work engagement. (b path)	0.23 (b path)	
Indirect effect (a ∗ b) of congruence via WE		0.06^∗∗∗^ (indirect effect)
99% bootstrapped CIs for indirect effect (ab)		(0.03, 0.10)

*Standardized coefficients are reported. Bias corrected confidence intervals (CIs) in 20,000 bootstrap samples are reported. Control variables include gender similarity, age similarity, education similarity, and tenure with the supervisor. WE, work engagement. ^∗^p < 0.05, ^∗∗^p < 0.01, ^∗∗∗^p < 0.001.*

The block variable for leader–follower PDO congruence is positively related to trust in supervisor (*R* = 0.33) and work engagement (*R* = 0.20). In addition, trust in supervisor and work engagement are positively correlated with task performance (*R* = 0.24; *R* = 0.23), and the effect of the block variable on task performance is significant when trust in supervisor and work engagement is taken into account. Bias-corrected bootstrapped confidence intervals of the indirect effect of leader–follower PDO congruence on task performance is 0.078 (CI95% = [0.04, 0.13]), which excludes zero (through trust in supervisor); and the indirect effect of leader–follower PDO congruence on task performance is 0.057 (CI95% = [0.03, 0.10]), which excludes zero (through work engagement). In addition, after adding mediator variable, we took task performance as the dependent variable for regression again (Model 10 and Model 11). The specific coefficients of mediator variable are significant as shown in Table 3 (for trust in supervisor: *R* = 0.24, *P* < 0.001; for work engagement: *R* = 0.28, *P* < 0.001). Overall, these findings lend support for H4 and H5.

### The Mediating Effect of Trust in Supervisor on the Relationship Between Leader and Follower Power Distance Orientation Congruence/Incongruence and the Work Engagement

Regarding the mediating effect of leader and follower PDO congruence/incongruence and the work engagement via trust in supervisor, the block variable for leader–follower PDO congruence is positively related to trust in supervisor (*R* = 0.33). In addition, trust in supervisor is positively related to work engagement (*R* = 0.43). Bias-corrected bootstrapped confidence intervals of the indirect effect of leader–follower PDO congruence on task performance is 0.14 (CI95 = [0.10, 0.20]), which excludes zero. moreover, we took work engagement as the dependent variable for regression after adding mediator variable (Model 7). The specific coefficients are significant as shown in Table 3 (*R* = 0.43, *P* < 0.001) support for H6.

In addition as Table 7 shows, after examining the indirect effects of trust in supervisor and work engagement, respectively, this study tested the chain mediating effect of trust in supervisor and work engagement on the relationship between leader-subordinate PDO congruence and task performance. Results of bootstrap with 20,000 draws showed that bias-corrected bootstrapped confidence intervals of the chain mediating effect of leader–follower PDO congruence on task performance is 0.03, (CI95 = [0.02, 0.05]).

**Table 7:** Results for indirect effects of leader and follower power distance orientation congruence on work engagement.

Variable	Mediator trust in supervisor	Work engagement
Coefficient of the block variable	0.33 (a path)	0.05^∗∗∗^ (direct effect)
Trust in supervisor	0.43 (b path)	
Indirect effect (a∗b) of congruence via TS		0.14^∗∗∗^ (indirect effect)
99% bootstrapped CIs for indirect effect (ab)		(0.10, 0.20)

*Standardized coefficients are reported. Bias corrected confidence intervals (CIs) in 20,000 bootstrap samples are reported. Control variables include gender similarity, age similarity, education similarity, and tenure with the supervisor. TS, Trust in Supervisor. ^∗^p < 0.05, ^∗∗^p < 0.01, ^∗∗∗^p < 0.001.*

## Discussion

According to Leung et al. (2005), it is more persuasive to study cultural dimensions at an individual level than at a national level. The influence of leader–follower power distance-orientation congruence on subordinate task performance is examined in this study from the individual level based on implicit leadership theory and 526 matching data from supervisors and subordinates. The congruence of leader–follower PDO was tested using polynomial regression and response surface methodology, and the mediating effect of trust in supervisor and work engagement was tested by block variable approach recommended by Edwards and Cable (2009). The results indicated leader–follower PDO congruence affect employee task performance through trust in supervisor and work engagement, and also affect work engagement through trust in supervisor. In a situation of congruence, low–low ratings congruence in leader-subordinate PDO will lead to better task performance and work engagement than the high–high congruence. In addition, in a situation of incongruence, subordinate task performance and work engagement is higher when a leader’s PDO is lower than a follower’s PDO.

### Theoretical Contribution

This research has vital theoretical significance. First, it reveals the main effect of PDO congruence. Most studies of power distance focus on its moderating effect, but overlook its main effect of congruence. From the perspective of congruence, this study uses polynomial regression and surface response analysis to comprehensively examine the impact of PDO congruence in leader-subordinate duality. Second, the study reveals the cognitive path of the influence of PDO congruence on individual performance. Based on the trust model of Mayer et al. (1995), the mediating role of trust in supervisor in the relationship between leader–follower PDO congruence and subordinate task performance and work engagement is analyzed, thus enriching the research on the mediating mechanism of PDO congruence. The study also contributes to the practical research of trust. Most current research considers supervisor trust solely from the perspective of leaders or employees, such as examining the effects of various leadership styles on employee trust in supervisor, but fail to assess whether the individual characteristics of leaders and subordinate employees together have an impact on supervisor trust in their dyadic relationships. The impact of four models of leader–follower PDO congruence on trust in supervisor are analyzed in detail. The results show that an increase in superior-subordinate power distance-oriented congruence has a negative impact on trust in supervisor, while in the case of incongruence, subordinate trust in supervisor and work engagement is higher when a leader’s PDO is lower than a follower’s PDO. Trust in supervisor also mediates the influence of the leader–follower PDO congruence on employees’ work engagement and task performance.

### Management Implications

The purpose of this research is to examine the mechanism of leader–follower PDO congruence, which provides effective management inspiration for enterprises, and to reduce the level of management resulting from the cognitive differences of power distance between superiors and subordinates. Based on these conclusions, we propose the following management recommendations. First, leadership style with low PDO should be encouraged and developed. The results of this study show that leader–follower PDO congruence has a positive impact on subordinates trust in leader, work engagement, and task performance. Particularly in cases of congruence, low–low ratings congruence in leader-subordinate PDO will lead to more trust in supervisor and work engagement of subordinates. This requires organizational leaders to engage in and carry forward an approachable leadership style in daily management, and to mobilize staff to participate in decision-making and to actively make suggestions. Studies have shown that as the central to the competitiveness of enterprises, knowledge workers always pursuit of equality and the weakening of authority, which make them more inclined to engage in equal dialog and communication with leaders (Janz et al., 1997). The approachable leadership style through low power distance-oriented leader will be more easily recognized and respected by knowledge workers, providing them with a sense of intimacy and thus a sense of trust and belonging, which will have a positive impact on organizational performance. In addition, good images of corporative leaders should be developed and established. The results of the study demonstrate that trust in supervisor mediates the impact of leader–follower PDO congruence on task performance, and further mediates its impact on work engagement. The positive impact of trust in supervisor cannot be underestimated. The enterprise leaders should cultivate their own trustworthy traits, through words and deeds, and constantly improve their reliability in terms of contact with employees. Employees’ recognition of leaders will thus increase, and stimulate their enthusiasm for work, which results in better employee performance. It is also necessary to help managers at all levels establish the high-quality leadership image, establish the necessary training and publicity material, and effectively develop corporate personality marketing.

### Limitations and Recommendations

One potential limitation of this research is that the two mediating variables of this study (trust in supervisor and work engagement) were simultaneously measured in the second stage of the survey. The relationships among the variables are likely inflated (Crampton and Wagner, 1994). Future research can be conducted in lab-setting for data collection or longitudinal research can be designed to improve the reliability of the research and effectively avoid Common Source Bias. In addition, this study only reveals the mediating roles of trust in supervisor and work engagement between leader–follower PDO congruence and task performance. The research can be expanded by examining other mediating mechanisms. For example, according to the role expectation theory, the congruence of power distance between supervisors and subordinates may increase subordinates’ satisfaction of their role expectations, which in turn influence their work behavioral outcomes. Finally, this study does not consider moderating variables in revealing the mediating effect of leader–follower PDO congruence. Task performance is not only influenced by leadership-subordinate power distance-oriented congruence, but also by external factors, such as job characteristics. Future research can explore the boundary condition of situational factors to enrich the results.

## Author Contributions

YB wrote the manuscript and collected the data. SL, JL, and ZG helped with the paper revision and acquisition. YZ edited the method part. CD and ZG helped with the final revision. All authors listed have made a substantial, direct and intellectual contribution to the work, and approved it for publication.

## Conflict of Interest Statement

The authors declare that the research was conducted in the absence of any commercial or financial relationships that could be construed as a potential conflict of interest.
